# Allogenic Bone Graft in Dentistry: A Review of Current Trends and Developments

**DOI:** 10.3390/ijms242316598

**Published:** 2023-11-22

**Authors:** Michał Ciszyński, Sebastian Dominiak, Marzena Dominiak, Tomasz Gedrange, Jakub Hadzik

**Affiliations:** Department of Dental Surgery, Faculty of Dentistry, Wroclaw Medical University, Krakowska 26, 50-425 Wroclaw, Poland

**Keywords:** bone, allograft, derivatives, osteoinductive, osteogenic, properties, dentistry

## Abstract

In an effort to prepare non-autologous bone graft or biomaterial that would possess characteristics comparable to autologous bone, many different allogenic bone derivatives have been created. Although different existing processing methods aim to achieve the very same results, the specific parameters applied during different stages material preparation can result in significant differences in the material’s mechanical and biological properties The properties, including osteoconductive, osteoinductive, and even osteogenic potential, can differ vastly depending on particular preparation and storage techniques used. Osteogenic properties, which have long been thought to be characteristic to autogenic bone grafts only, now seem to also be achievable in allogenic materials due to the possibility to seed the host’s stem cells on a graft before its implantation. In this article, we aim to review the available literature on allogenic bone and its derivatives as well as the influence of different preparation methods on its performance.

## 1. Introduction

Bone resorption following teeth loss can lead to a significant reduction in the volume of surrounding bone, caused by the lack of periodontium, which would, normally, provide additional vascularization and stimulate the alveolar bone to maintain its structure [1]. Reduced bone dimensions prevent achieving satisfactory functional and aesthetic outcomes after placing dental implants. Despite the effort to produce implants of reduced height and width, the need for repairing lost bone volume persists. The deficit of bone can only be restored through bone grafting, which has now become an integral part of oral surgery, especially considering patients’ rising awareness and expectations towards this type of treatment [2]. Estimates suggest that up to a half of all dental implant procedures performed will involve the use of bone grafts [3].

A bone graft is defined as a tissue capable of promoting bone healing, transplanted into a bony defect, either alone or in combination with other materials. To date, many different bone regeneration materials, as well synthetic and natural, have been used both in orthopaedic and maxillofacial surgery [4]. An ideal bone graft should be biocompatible, resorbable, sterile, and easy to handle. It should provide mechanical support and stimulate osteo-regeneration through three crucial characteristics: osteogenesis, osteoconduction, and osteoinduction [4,5]. Osteogenesis is the process of forming bone by osteoblasts and their precursors retained in the graft after harvest and transplantation. Osteoinduction, in turn, is the ability of a grafting material to encourage the host’s organism through different biochemical factors to produce new bone tissue. One of the mechanisms is stimulating the host’s mesenchymal stem cells to differentiate into preosteoblasts and further osteoblasts, thus promoting osteogenesis. The last characteristic of an ideal bone graft, osteoconduction, refers to the ability of a material to provide a scaffold for the ingrowth of vessels, osteoblasts, and the host’s stem cells. Some authors propose osseointegration, defined as the ability of a biomaterial to bond chemically to the surface of the host’s bone without the intervening fibrous tissue layer, as the fourth fundamental trait [2,4,6,7]

Aside from bone regeneration and bone substitute materials, soft tissues can also be augmented using different materials, including autogenous and xenogenic [8]. Blood-derived autogenous materials, such as Plasma Rich in Growth Factors (PRGF), Leukocyte and Platelet-Rich Fibrin (L-PRF), Advanced Platelet-Rich Fibrin (A-PRF) and Concentrated Growth Factors (CGF), are worth mentioning as they can significantly improve healing and are widely used in many fields of modern dentistry [9,10,11].

Bone healing is important not only in cases where a clinician needs to increase the amount of bone in a particular area, but also in cases of fractures. Osteosynthesis can be achieved through a conservative or surgical approach, and using non-resorbable and resorbable materials applied to stabilize separated fragments and to allow for bone healing [12]. Particular resorbable biomaterials can be welded into the fractured bone using ultrasound, which allows for a more predictable and comfortable way of placing osteosynthesis plates [13].

Monitoring and understanding the remodeling of bone surrounding dental implants is essential to achieving improvements in their survival rates. This seems to be even more important in the case of implants placed in grafted bone, for example, after a sinus lift procedure, as the bone’s quality can often be inferior to the patient’s own, ungrafted tissue. Measuring the grey values of cone beam-computed tomography can be used as a non-invasive, qualitative method able to support follow-ups and closely monitor bone density [14,15].

The title of “gold standard” in bone grafting procedures belongs to autogenous grafts, as they are the only group to have osteogenic, osteoinductive and osteoconductive properties. Unfortunately their limited availability and possible complications at the donor site after harvesting determine the need for different grafting materials. Despite the widespread appliance of grafting procedures, no other material has osteogenic potential [6,7]. In grafting procedures, clinicians have not only used bone harvested from donors, but also bone substitute materials like hydroxyapatite [16]. Despite the availability of different grafting materials, alternatives are often sought in order to avoid performing bone augmentation procedures where not absolutely necessary. Examples include the use of shorter dental implants instead of performing sinus lift procedures [17].

Allogeneic bone can have both osteoinductive and osteoconductive properties and can be obtained from live or cadaveric donors of the same species, but with different genotypes. Fresh or frozen allogeneic bone tends to display more favorable osteoinductive and mechanical properties, but is also associated with shorter usability span, and the risk of disease transmission or host immunogenic response occurring [4,18]. Due to the above, the need emerged to develop processing and storage techniques that would minimize risk and lengthen shelf life. Unfortunately, allogenic bone’s properties tend to differ greatly depending not only on the sterilization methods applied, but also histological type. Available allograft materials can include fresh, frozen or freeze-dried forms and can either contain cortical, cancellous or cortico-cancellous bone tissue. Clinicians can choose whole bone segments, chips, wedges, pegs and powder. Aside from the above, allograft’s processing has led to obtaining clinically usable derivatives such as the Demineralized Bone Matrix (DBM), Autolyzed, Antigen-Extracted, Allogeneic (AAA) bone, and Bone Decellularized Extracellular Matrix (dECM, bone dECM). In this article we aim to discuss the differences between different allograft bone materials and the current state-of-the-art-concerning allogenic bone [4,19,20,21].

## 2. Different Allogenic Bone Materials

**Figure 1:**
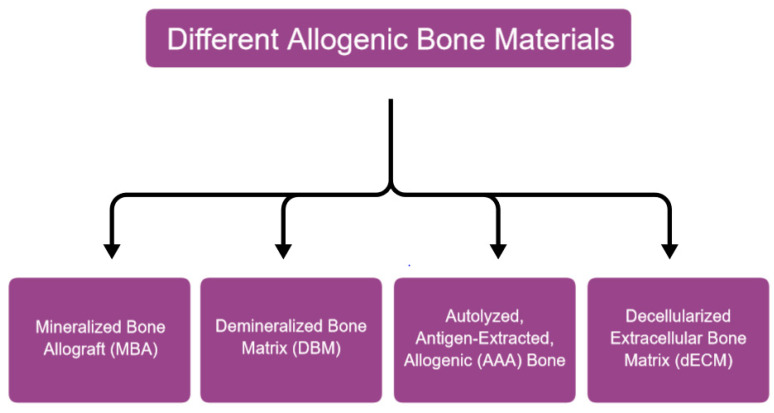
Different allogenic bone materials.

While reviewing available literature on allogeneic bone, one can encounter some disturbing discrepancies resulting from the lack of unambiguous classification of allograft types. In existing scientific papers, there is information about Mineralized Bone Allograft (MBA), Fresh-frozen Bone (FFB) Allograft, Freeze-Dried Bone Allograft (FDBA), Mineralized Freeze-Dried Bone Allograft (MFDBA), Decalcified Freeze-Dried Bone Allograft (DFDBA), Autolyzed, Antigen-extracted Allogenic bone (AAA bone), the Demineralized Bone Matrix (DBM), and the Decellularized Extracellular Matrix (dECM). In fact, FDBA and MFDBA seem to be the same thing under two names, while, in fact, being MBA after freeze drying (lyophilization). Similarly, the names DFDBA and DBM appear to have been used interchangeably. Arguably MBA, DBM, AAA bone and dECM are separate allograft derivatives and will be described as such (Figure 1). A comparison of different properties of discussed materials can be seen in Table 1.

### 2.1. Mineralized Bone Allograft

The most basic form of allogeneic bone is fresh, unprocessed bone block. It can be then frozen (FFB), lyophilized (FDBA also known as MFDBA), or grafted immediately. As allograft processing techniques reduce its mechanical strength, fresh, unprocessed allograft has superior characteristics compared to processed bone. Unfortunately, for fear of possible disease transmission and host immunogenic response, fresh allografts are not very popular nowadays [4,7,21].

Similarly to the so-called fresh-frozen bone, freeze-dried grafts display prolonged shelf-life. During the lyophilization process, almost all water is removed from the tissue, further reducing its mechanical strength and destroying osteogenic cells. As Major Histocompatibility Complex class I molecules can be found on osteoblast surface, this also leads to the reduction of immunogenicity. Disease transmission risk is also reduced [7].

The mineralized allograft’s properties differ depending on whether it consists of cortical or cancellous bone tissue, or both. In general, authors seem to agree that both cortical and cancellous allograft can be osteoconductive, with cortical bone being characterized by having a more overall structural strength and better mechanical properties, which leads to a more favorable osteoconductive effect. Cortical bone is also more dense and displays slower resorption rates. Cancellous bone alone seems not to be mechanically resistant enough for most clinical appliances, but the presence of large intertrabecular space accounts for faster and more complete revascularization and incorporation after grafting. It is also worth noting that frozen cortical allograft’s structural strength, depending on preparation methods, usually far exceeds those of freeze-dried cortical tissues. Because of the above, cortical allograft is usually applied in the form of a whole bone segment, block, or pieces, while cancellous allograft’s available forms include chips, wedges, pegs, and powder. Neither of the two types of bone have osteogenic properties. For joined benefits of both bone types, allograft bone blocks often consist of cancellous and cortical bone. The compressible cancellous layer allows for achieving the precise fit of a graft, while the cortical bone provides mechanical strength and protection in the early stages of healing [4,19,22,23].

One of the early researchers, Urist, recognized osteoinductive properties to be dictated by many factors, including the presence of Bone Morphogenetic Proteins (BMPs) [24]. BMPs are most commonly located in cortical bone, which would suggest that cortical bone allografts should have better osteoinductive properties compared to the cancellous one. This appears not to be true, as most researchers indicate the contrary, claiming that cancellous bone can have some osteoinductive properties, while cortical alone cannot [4,19]. Nonetheless, different authors seem to agree that osteoinductivity is increased when the bone’s mineral component is reduced, increasing the availability of the extracellular matrix [4,19,25]. Modern research on osteoinductivity, however, indicates that it can occur not only due to the presence of BMPs, but also non-collagenous proteins (NCPs) such as bone sialoproteins, osteocalcin, osteopontin and proteoglycans. Moreover, complete demineralization of bone matrices results in lower osteogenic differentiation compared to mineralized or partially demineralized grafts. Specific minerals have been shown to also play a crucial role in achieving osteoinductivity. Magnesium is necessary for production of ATP, DNA, RNA and protein. Zinc enhances osteoblast differentiation and proliferation and lowers osteoclastic activity. Strontium inhibits osteoclast differentiation and activates calcium-sensing receptors which also stimulate osteoblast proliferation. Copper, in turn, enhances angiogenesis [26].

Unprocessed allografts have a very limited shelf-life, thus are often frozen or freeze-dried to make storage less problematic. Freezing of bone tissue at temperatures between −20 °C to −147 °C has been shown not to reduce its properties. Lyophilization (freeze-drying) can, in turn, cause damage to the collagen fibers, which leads to a reduction of mechanical properties of the processed tissue. This reduction is worsened when gamma irradiation is used together with lyophilization. Animal studies suggest that although the reduction of mechanical strength is significant (30% reduction of compressive strength, 41% reduction of bending strength and 60% reduction of torsional strength), rehydrating the bone before grafting can lead to partial recovery of the initial physical properties [27].

The effect of terminal sterilization of allogenic bone on its properties has been questioned as well. Research has shown that irradiation had minimal effects on the elastic modulus at all tested levels. Cortical bone does, however, become increasingly more brittle when treated with higher doses of radiation. A dose of 3 mrad leads to an overall strength reduction of 10–20%, but the bone becomes significantly more brittle (60% less energy can be absorbed before breaking). Again, the combination of gamma irradiation and lyophilization has been shown to cause more damage than either treatment alone. Cancellous bone is reported to be much more resistant to gamma irradiation (no measurable effects after treating tibial cancellous blocks with a dose of up to 5.1 mrad). In general, it is agreeable that irradiation negatively alters properties of bone tissue. Over time, lower and lower doses of irradiation have been shown to provide sufficient sterilization of bone samples [27,28,29,30].

Aside from the reduction in the mechanical strength of a graft, high-dose irradiation also has a detrimental effect on its osteoconductivity and osteoinductivity. Ethylene oxide, despite having been extensively used for the sterilization of allogenic bone, is known to significantly compromise the mineralized graft’s incorporation compared to tissues treated with hydrogen peroxide, or those that have been irradiated [27].

Another remarkable aspect is the possible immune response to the transplanted tissues, causing their rejection. In order to avoid it, a properly processed mineralized allogenic bone should contain as little as possible of the remaining donor’s cells or cellular debris. This can be achieved through washing with water, ethanol, or mild solvents [18,27].

### 2.2. Demineralized Bone Matrix (DBM)

As demineralized bone allograft, more commonly known as the Demineralized Bone Matrix (DBM), is void of the mineralized matrix, the bone inductive biochemical factors of the bone’s extracellular matrix, including BMPs, NCPs and growth factors, become more bioavailable, and thus, DBM has superior osteoinductive features compared to mineralized allogenic bone, as well cortical and cancellous [4,19,27,31].

DBM, like other allografts, show no osteogenic ability. As aforementioned, its osteoinductive potential is much higher than that of conventional allografts, due to the increased availability of the bone’s extracellular matrix’s osteoinductive proteins. After grafting, DBM slowly releases BMPs, which further increases its osteoinductive potential. While all available DBM materials appear to have osteoinductive properties, there can be some differences between them [4,32,33,34]. It also displays osteoconductive ability, even though it is inferior to that of conventional, mineralized allograft. Unfortunately DBM itself lacks structural strength, which results in poor mechanical properties. Thus, it is often combined with other allografts, or even other grafting materials [4,22,35].

Original forms of freeze-dried DBM were difficult to handle. The material did not cohere, was not moldable and would disperse in a hemorrhagic environment [25]. This has led researchers to prepare DBM with other substances such as glycerol, starch, hyaluronic acid, collagen, and saline. Such preparation enables the hardening of DBM mixture, which results in the improved ease of use. Some studies, however, suggest that commercial formulations of DBM have less osteoinductive potential than DBM alone. Some studies, however, suggest that commercial formulations of DBM have less osteoinductive potential than DBM alone. This could possibly be due to the fact that adding a carrier effectively reduces the amount of DBM per unit volume, which can further be reduced by adding conventional allograft for improved mechanical properties [25,36]. DBM is commercially available in the form of putty, paste, blocks, particulates and powder.

As mentioned previously, in DBM, the extracellular matrix containing bone inductive proteins is more bioavailable, which greatly enhances its osteoinductive properties compared to those of mineralized allogenic bone. The particular type of demineralizing agent used to acquire DBM can, nonetheless, have an impact on its osteoinductive performance. Using a 0.5–0.6 m solution of hydrochloric acid and a 1:1 combination of formic and citric acid has shown to result in creating DBM with satisfactory osteoinductive properties. The use of hydrochloric acid together with alcohol has, however, caused DBM to not be osteoinductive. Similarly, the same has been conducted with nitric, nitrous, acetic and lactic acids, which also fail to maintain the bone’s osteoinductive properties. To increase the effectiveness of hydrochloric acid, sonication of 20,000 cycles per second was tested, but it was reported to negatively affect the osteoinductivity as well. The use of ethylenediaminetetraacetic acid (EDTA) as a chelating agent was shown not to achieve complete demineralization and thus reduce DBM’s performance. Fat-removing organic solvents, such as ether, acetone, or hexachlorophene, neither affect or improve osteoinductivity [27]. Some techniques of preparation involving the use of alcohol, lactic acid, acetic acid, or nitric acid reduce osteoinductive potential [4,19].

Aside from the choice of solution used for demineralization, the time during which allogeneic bone is exposed to, it also plays a crucial role. Remaining calcium content must be reduced to at least 40% before the extracellular matrix is exposed enough to allow for a strong osteoinductive effect [27].

Other actions taken during DBM’s production that have been tested include the use of antibiotics, high and low temperatures, and lyophilization. Oxytetracycline, erythromycin, streptomycin, chloramphenicol, and penicillin do not reduce the osteoinductive potential. In contrast, excessive heat (70–100 °C), as well as using more than three freezing and thawing cycles, have been shown to have a detrimental effect. Lyophilization and freezing DBM at −4 or −70 °C can be used so as to increase shelf-life without affecting osteoinductivity [27].

No general consensus seems to have emerged concerning whether sterilizing DBM with gamma irradiation in doses eliminates the osteoinductive response. Some researchers suggest that the dose of 2.5 mrad does not destroy DBM’s osteoinductive potential [37]. The same applies to the use of ethylene oxide in the conditions (temperature and duration) necessary to achieve the proper sterilization of DBM. The use of formaldehyde gas or glutaraldehyde solution also eradicates osteoinductive properties.

### 2.3. Autolyzed, Antigen Extracted, Allogenic (AAA) Bone

AAA bone is an allograft bone derivative created by incubating the Demineralized (completely or partly) Bone Matrix in neutral phosphate-buffered solutions, which leads to autolytic digestion of the bone’s cellular components. To avoid phosphate-buffer mediated activation of endogenous proteinases that would otherwise inactivate osteoinductive proteins, the addition of particular enzyme inhibitors is necessary. Examples include NaN_3_, iodoacetic acid, iodoacetamide, N-ethylmaleimide, phenylmethyl sulfonyl fluoride, benzamidine-hydrochloric acid, thimersol, and p-chloromercuribenzoate. Through this process, it is possible to obtain a fully osteoinductive biomaterial called AAA bone [20].

Similarly to other allogenic bone types, AAA bone requires sterilization before implantation. There have been cases of HIV transmission despite researchers’ belief that this product would not require terminal sterilization.

The structural strength of AAA bone depends on the extent of demineralization. Surface-demineralized samples displayed superior mechanical properties than completely demineralized ones. Nonetheless, all AAA samples show decreased mechanical properties than untreated, lyophilized bone. Surface-demineralized cortical bone’s mechanical strength is almost the same as that of untreated bone’s. It has been confirmed that AAA bone has osteoinductive properties. For the material to be osteoconductive and provide a scaffold for bone growth, only superficial layers of bone tissue should be demineralized [20].

The literature concerning AAA bone is scarce. Authors of this study put in the effort to find recent scientific papers on this topic, but researchers appear to have lost interest in this derivative of allogenic bone. This is likely due to the development of dECM, which appears very promising and is broadly discussed. The latest mention of AAA bone found is from December 2004 and refers to orthopaedics, not oral surgery [38].

### 2.4. Decellularized Extracellular Matrix (dECM)

The term Decellularized Extracellular Matrix refers to various types of allogenic biomaterials made of human or animal tissue after the removal of cellular components that would, in normal conditions, promote immunogenicity. It has been shown that whole organs such as lungs or the heart can be decellularized for future replacement, and not only bone tissue. The biological properties and physicochemical signals of dECM can be retained despite the preparation process, which makes dECM a proper 3D scaffold for subsequent stem cell seeding [39].

dECM can be obtained in the form of a hydrogel, particles, and cell-laid matrix (after recellularization). In addition, it has also been used as a bioink in 3d-bioprinting [6,39,40].

dECM can be, in general, obtained in two ways: it can be tissue-derived or cell-derived. Tissue-derived bone dECM is bone derived from an animal or a human, alive or cadaveric. It is then subjected to the process of decellularization, which is discussed below. Cell-derived bone dECM is created after culturing mesenchymal stem cells in vitro. To achieve the desired shape of the resulting dECM, the culturing is usually conducted on a scaffold, such as hydroxyapatite [39,41].

Tissue-derived bone dECM retains tissue-specific memory and can thus promote cell differentiation. It also keeps the complicated macro- and microstructural architecture, which influences cell behavior and improves osteoinductivity. Bone dECM also preserves immunomodulatory cytokines (TGF-β, BMPs, bFGF), regulating the pro-inflammatory response. The mechanical characteristics of tissue-derived bone dECM are also favorable compared to those of cell-derived bone dECM. Cell-derived dECM, in turn, has a lower risk of disease transmission [39].

As for the methods of preparing dECM, no gold standard has emerged. Physical, chemical and enzymatic treatments are conducted in different configurations in efforts to achieve the complete removal of cellular components without damaging the ECM’s structure and properties.

Physical methods include freeze–thawing cycles, supercritical carbon dioxide (SC-CO2) treatment, osmotic lysis, sonication, and electroporation. Freeze-drying in nitrogen and thawing in buffer solutions have been used for bone tissue and shown to disrupt cell membranes and cause cell lysis, and additionally cause the cells to detach from ECM. It is the most common physical measure taken to decellularize bone allograft and allows to maintain ECM’s integrity. Repeated cycles have been used to decellularize, as well as tissue-derived and cell-derived ECM. The efficiency of such a process depends on the cooling/thawing rate, number of the cycles, processing time and, of course, temperature. After freeze–thaw cycles, the cellular components need to be removed through subsequent treatments [40,41,42,43].

Supercritical carbon dioxide treatment seems very beneficial, as it is compatible with biomaterials, leaves no toxic waste, and was shown to efficiently remove cells at 30 MPa pressure and 50  °C temperature. Rapid pressure reduction is obligatory for proper decellularization. Some authors claim this method does not require further sterilization and causes no reduction to the mechanical and structural strength of dECM. It also is a relatively short-timed procedure [44].

Chemical decellularization is conducted through different acids, bases, and detergents. Basic solutions, such as sodium hydroxide and calcium hydroxide, are harsher and may damage the ECM structure, thus reducing its structural stability. Acidic solutions, such as peracetic acid, acetic acid, and hydrochloric acid, may, in turn, be less effective. Bone, as a hard tissue, usually requires stronger solutions for proper decellularization [37,40]. Detergents can be divided into ionic, nonionic and zwitterionic. Nonionic detergents are weaker and damage the cell membrane while preserving protein–protein interactions. Ionic detergents, such as sodium dodecyl sulfate (SDS), are stronger and also denature the proteins. Zwitterionic detergents are characterized by medium strength and are mainly used for thin tissues. Like in the case of basic solutions, stronger detergents are preferred in the case of bone tissue [40,45].

Enzymatic methods include the use of proteases and nucleases and are usually applied following chemical decellularization to complete the process. Trypsine, deoxyribonuclease and ribonuclease are used in the process [40].

The existing literature on the mechanical and biological properties of bone dECM seems to be scarce. In one study, the authors claim that decellularized human radius bones had significantly worse mechanical properties. Compressive strength did not change for ulna bones, while humerus bones retained their flexural strength after decellularization. Regardless of bone type, after decellularization, all bones fractured in a different manner, and brittle fractures occurred, which are characteristic for a significant stress drop at a critical compressive stress value. The authors highlight that radius bone has been chemically treated for twice as long as ulna and humerus, which might be the reason for the significant reduction of mechanical strength. The reduction is associated with damage due to collagen [45].

As was shown in a systematic review by Amini and Lari, clinicians and researchers apply different processing techniques in various configurations [46].

To verify the extent of decellularization, the amount of double-strand DNA (dsDNA) is measured before and after the process. As indicated by Crapo et al. in 2011, one mg of dried ECM should contain no more than 50ng dsDNA, and the remaining dsDNA chains should be no longer than 200 base pairs. The remaining dsDNA should not be visible in DAPI or hematoxylin and eosin staining [40,47].

After preparing bone dECM, it requires postprocessing to remove toxic components and improve biocompatibility. Sterilization and disinfection techniques include irradiation with gamma rays and electron beams, ethylene oxide gas sterilization, antibiotic disinfection, and peracetic acid treatment. Sterilization with gamma rays and electron beams can damage DNA and proteins of microorganisms. However, just like in the case of mineralized allogenic bone, it can alter dECM’s characteristics. Ethylene oxide does not damage dECM, but it produces toxic residues. Antibiotic disinfection hardly affects the characteristics of dECM, but it has no effect on viruses and spores, which makes it insufficient alone. Peracetic acid treatment can achieve antibacterial effect as well, and products of its decomposition are not toxic; however, it might have a detrimental effect on the properties of dECM scaffolds [39].

dECM’s mechanical properties and stability can be enhanced through the process of cross-linking. Glutaraldehyde is frequently used for crosslinking collagen-based materials. Less cytotoxic alternatives include glyoxal and genipin. dECM can also be surface-modified to increase pore density and improve cell infiltration. Methods of surface modification aiming to increase pore density and improve cell infiltration include laser beam modification, solvent casting, and electrospinning modification. The methods are still being developed, and not all of them have been successfully applied in the case of bone dECM in particular [39].

Finally, after obtaining sterile bone dECM, it is possible to recellularize it with future host stem cells. For bone tissue, specifically, mesenchymal stem cells (MSCs), with their ability to differentiate into osteoblasts, seem like a promising possibility. It was shown that MSCs can be obtained from the pulp of removed human teeth [48,49,50].

The ability of seeding immunologically compatible stem cells on a bone dECM scaffold seems to be a particularly promising perspective, as it could allow clinicians and patients to benefit from not only osteoconductive and osteoinductive, but also osteogenic potential [39].

It was shown that the bones of old donors is better than bone from young donors in supporting stem cells’ osteogenic differentiation. Young donor’s MSCs, however, have proven to possess superior differentiation capacity [51].

## 3. Discussion

The goal to create an ideal allogenic bone graft characterized by osteoinductive, osteoconductive and osteogenic potential has long been pursued by researchers. For allograft’s properties to be satisfactory, the history of processing and storage methods applied before intended use are equally as important as the bone used. Thus, belonging to the same category can, in fact, have different properties.

The fact that the seemingly same biomaterial can have varying properties depending on particular parameters of processing methods can have a detrimental effect on the success rate and clinical effects. Most tissue banks offering allogenic bone do not specify all the physical and chemical processes that were applied during the graft’s preparation, not to mention that exact values of parameters like temperature, pressure, time and other significant variables are not available. The clinicians seem to be unaware of potential differences between materials from different tissue banks and, perhaps, even from within one tissue bank. This can lead to major discrepancies in the quality of biomaterials used during surgeries performed worldwide, and to surgeons now knowing the exact properties of the material being used.

Available bone derivatives differ greatly, but, unfortunately, almost none of them possesses the pursued osteogenic properties. Fresh, unprepared allogenic bone does contain cellular components and could be osteogenic, but is hardly used nowadays for fear of disease transmission and host immunogenic response. The methods applied to prolong shelf-life and reduce immunogenicity destroy cellular components and thus strip the material of osteogenic properties. The golden standard of bone augmentation being autogenous bone graft has major drawbacks. It requires additional surgery at the donor site, and is associated with more complications after operation. The possibility of seeding the host’s stem cells on dECM after the whole preparation process can change how allogenic bone is seen by clinicians worldwide. Allografts themselves may stimulate Toll-like receptors of the patient’s immune system and induce release of signaling molecules which boost the reparative immune response. The addition of the host’s stem cells may be the way to improve the healing process after bone regeneration. A. Mansour et al. managed to coat bioceramic material with bone extracellular matrix cells and improved regenerative properties of the scaffold-like material [52]. This can be a sign that allografts coated with patient’s stem cells may, in the future, be regarded as a new gold standard of atrophic bone regeneration.

However, an allogenic bone graft may induce a response from the immune system via Human Leukocyte Antigen (HLA) [53]. Contact with HLA antigens can occur during other transplantation procedures, blood transfusion, or pregnancy. Higher volumes of HLA antigens in serum can lead to prolonged healing or osteointegration time, or even the rejection of a bone transplant [54]. According to M. Piaia et al. [55], augmentation of the maxillary sinus for further implantation may be a cause for the sensitization to the HLA antigens. Thus, while planning bone augmentation with allogenic material, HLA presence should be taken into consideration, especially when treating patients where sensitization could occur before, or can interact with their other treatment. There is, however, no consensus whether the presence of HLA antibodies can be harmful for the graft integrations. In Redondo-Pachon et al., the study results show there is no big impact on the bone substitute survival depending on whether the antibodies are present or not [56]. On the other hand, in a different study, the presence of HLA antibodies appeared to correlate positively and there was a statistically significant difference between graft rejections in different groups [57].

## 4. Future Directions

It appears clear to the authors of the study that further research should revolve around the methodology of seeding stem cells on dECM and applying it clinically. Utilizing modern equipment, such as 3D printers, cone based computer tomography and intraoral scanners, could lead clinicians to be able to prepare precise-fitting allogenic bone blocks that would, thanks to the presence of stem cells, possess not only osteoconductive and osteoinductive, but also osteogenic properties. We suspect that this might eventually become the new golden standard of bone regeneration in dentistry.

Moreover, the authors agree that bone banks should aim to improve the availability and quality of information concerning a particular graft’s preparation methods.

## Figures and Tables

**Table 1:** Properties of different allogenic bone materials.

Bone Allograft Type	Mechanical Properties	Osteoconduction	Osteoinduction	Osteogenesis
Mineralized, cortical allograft	+++	++	−	−
Mineralized, cancellous allograft	+	+	+/− *	−
Demineralized Bone Matrix (DBM)	+/− **	+	++	−
Autolyzed, Antigen Extracted, Allogenic (AAA) Bone	+/− **	+/− *	++	−
Decellularized Extracellular Bone Matrix	++	++	++	+/− ***

* No clear consensus seems to emerge from the literature. ** Depending on the extent of demineralization and type of bone used (cortical/cancellous). *** Depending on whether the material has been seeded with mesenchymal stem cells after preparation.

## Data Availability

The data presented in this study are available on request from the corresponding author.
